# Recent Advances in Inflammatory Diagnosis with Graphene Quantum Dots Enhanced SERS Detection

**DOI:** 10.3390/bios12070461

**Published:** 2022-06-27

**Authors:** Seyyed Mojtaba Mousavi, Seyyed Alireza Hashemi, Masoomeh Yari Kalashgrani, Darwin Kurniawan, Ahmad Gholami, Vahid Rahmanian, Navid Omidifar, Wei-Hung Chiang

**Affiliations:** 1 Department of Chemical Engineering, National Taiwan University of Science and Technology, Taipei City 106335, Taiwan; jdkywt@gmail.com; 2 Nanomaterials and Polymer Nanocomposites Laboratory, School of Engineering, University of British Columbia, Kelowna, BC V1V 1V7, Canada; s.a.hashemi0@gmail.com; 3 Biotechnology Research Center, Shiraz University of Medical Science, Shiraz 71468-64685, Iran; masoomeh.yari.72@gmail.com (M.Y.K.); gholami@sums.ac.ir (A.G.); 4 Centre of Molecular and Macromolecular Studies, Polish Academy of Sciences, Sienkiewicza 112, 90-363 Lodz, Poland; vahid_rahmanian@live.com; 5 Department of Pathology, School of Medicine, Shiraz University of Medical Sciences, Shiraz 71468-64685, Iran; omidifarn@sums.ac.ir

**Keywords:** inflammatory, graphene quantum dots, SERS, detection

## Abstract

Inflammatory diseases are some of the most common diseases in different parts of the world. So far, most attention has been paid to the role of environmental factors in the inflammatory process. The diagnosis of inflammatory changes is an important goal for the timely diagnosis and treatment of various metastatic, autoimmune, and infectious diseases. Graphene quantum dots (GQDs) can be used for the diagnosis of inflammation due to their excellent properties, such as high biocompatibility, low toxicity, high stability, and specific surface area. Additionally, surface-enhanced Raman spectroscopy (SERS) allows the very sensitive structural detection of analytes at low concentrations by amplifying electromagnetic fields generated by the excitation of localized surface plasmons. In recent years, the use of graphene quantum dots amplified by SERS has increased for the diagnosis of inflammation. The known advantages of graphene quantum dots SERS include non-destructive analysis methods, sensitivity and specificity, and the generation of narrow spectral bands characteristic of the molecular components present, which have led to their increased application. In this article, we review recent advances in the diagnosis of inflammation using graphene quantum dots and their improved detection of SERS. In this review study, the graphene quantum dots synthesis method, bioactivation method, inflammatory biomarkers, plasma synthesis of GQDs and SERS GQD are investigated. Finally, the detection mechanisms of SERS and the detection of inflammation are presented.

## 1. Introduction

When tissue is damaged by bacteria, trauma, chemicals, heat, or other phenomena, several substances are released from the damaged tissue that cause very serious secondary changes in the tissue. This series of tissue changes is called inflammation. Inflammation is basically a protective response that fights the cause of cellular damage (e.g., germs or toxins) and the consequences of that damage, i.e., necrotic cells and tissues [1,2,3,4]. Inflammation causes hypersensitivity reactions to insect bites, drugs, and toxins, as well as some chronic diseases, such as rheumatoid arthritis, atherosclerosis, and pulmonary fibrosis [5,6,7,8]. Inflammation is a complex process that begins with tissue damage caused by endogenous factors, such as tissue necrosis and bone fractures, or exogenous factors, such as mechanical, physical, biological damage (like infection with microorganisms or immunological responses, such as hypersensitivity reactions), and is accompanied by the invasion of inflammatory cells into the inflamed area [9,10]. Graphene quantum dots are a new generation of quantum dot structures. This new generation of carbon compounds contains a large number of functional groups and is synthesised in very small dimensions that exhibit a variety of other properties of carbon-based materials. Graphene quantum dots generally have a sheet structure of less than 10 nm in size and have received much attention in recent years due to their unique chemical and physical properties [11,12]. Compared with semiconductor quantum dots, graphene quantum dots are characterised by high water solubility, good photoluminescence properties, biocompatibility, optimal accessibility, easy surface functionalization, high stability, and low toxicity [13,14]. Thus, GQDs effectively reduce hyperinflammation by regulating immune cells, suggesting that they can be used as promising diagnostic agents to diagnose inflammation [15,16]. Surface-enhanced Raman spectroscopy (SERS) has also attracted great interest in various fields, such as medicine and analytical chemistry, due to its unique properties—sensitivity to single molecules on the surface, multiplexing potential, and fingerprinting capabilities [17,18,19,20]. One of the most important substrates of SERS, which includes quantum dots, has a wide range of applications in inflammation diagnosis, biological and chemical imaging, and labelling due to the plasmonic resonance properties of the surface-dependent local area, the chemical richness of the core-shell, and the compatible size [21,22,23,24,25]. In addition, graphene quantum dots also serve as a building block for an atomically flat SERS substrate, in which a much more uniform Raman signal can be obtained [26,27,28]. Graphene quantum dots (GQDs) have more accessible edges and larger specific surface areas than conventional graphene sheets, resulting in more efficient adsorption of target molecules [29,30,31].

This review study aims to explore recent advances in inflammation diagnostics using graphene quantum dots to improve the detection of SERS and highlight future areas of work in this field. In addition, the graphene quantum dots, bioactivation method, GQD synthesis method, inflammation biomarkers, plasma synthesis of GQDs, and SERS GQD were investigated. In addition, the detection methods of SERS and the detection of pro-inflammatory substances were evaluated.

## 2. Graphene Quantum Dot

**Figure 1:**
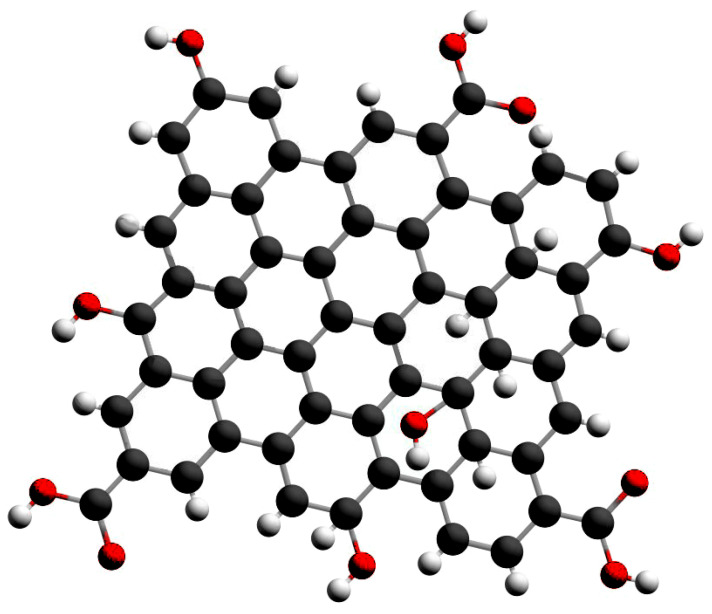
Graphene quantum dot. ( 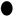: Carbon, 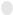: Hydrogen, 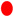: Oxygen).

Graphene quantum dots have attracted the attention of many researchers due to the crystalline structure of a single or a small amount of crushed graphene. These nanoparticles are a small lattice structure of honeycombs of carbon atoms that are less than 10 nanometers in size. Graphene quantum dots (GQDs) are, by definition, a type of quantum dot material with a graphene-derived property and carbon dots that can be placed in the form of very small graphene pieces (Figure 1). The carbon dots exhibit strong optical absorption in the UV region (260–320 nm) with an extended sequence in the visible and infrared regions. In addition, GQDs are semiconductor quantum dots with excellent light stability, biocompatibility and low toxicity, good electron mobility and good chemical stability, small size, electrochemical luminescence, photocatalyst capabilities, and are suitable for the fabrication of multiple sensors and bioimaging [32,33,34,35,36]. GQDs are less toxic than graphene oxides and have no obvious toxicity in the body, so GQDs have attracted much attention in biological applications, especially in the field of biopharmaceuticals [37,38]. Numerous groups have explored recent advances in graphene quantum dots for the construction of various sensors, including electron sensors, photoluminescence sensors (PL), electrochemical sensors, electrochemical luminescence sensors, PL-based high-conversion sensors, and surface-enhanced Raman spectroscopy (SERS) [39,40,41,42]. GQDs have become a prominent substance for the design of SERS due to their outstanding properties and model assumptions, such as high electron transfer rate, higher biomolecule loading, fast transduction, larger surface areas, easy surface functionalization, and inimitable electrocatalytic properties. These GQD enhanced SERS have been utilised for the detection of nucleic acids, amino acids, bioflavonoids, vitamins, small molecules, biomarkers, and heavy metal ions with remarkable properties [43].

### 2.1. Method of Synthesis GQD

**Figure 2:**
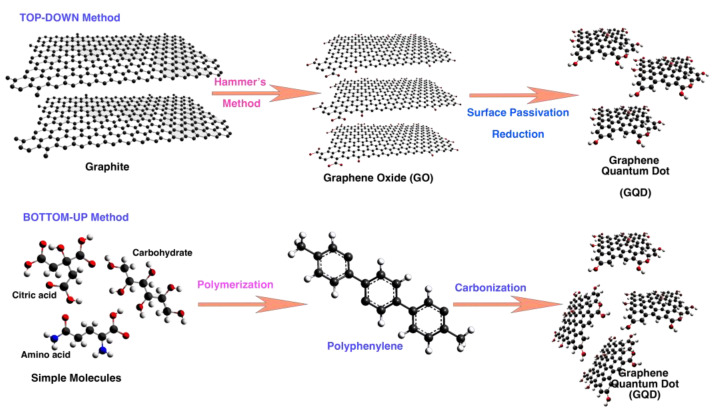
Schematic diagram representing the top-down and bottom-up approaches for the synthesis of GQDs.

Graphene quantum dots are synthesized to optimize the size of quantum dots by two methods: top-down and bottom-up (Figure 2). In the top-down method, bulky carbon, graphite, and graphene materials are converted into graphene quantum dots, while in the bottom-up method, organic molecules are used as the carbon source. The disadvantages of the top-down method are the difficulties in controlling the size distribution and morphology of the produced particles. In contrast, the properties of the produced nanoparticles can be well controlled by the bottom-up method. In general, the optical properties of graphene nanodots depend on the size and the effect of quantum confinement, which changes the density and the nature of sp2 sites. Therefore, the energy of these nanoparticles changes with the size of the gap [44,45,46,47]. Functionalizing the surface and doping the GQDs with other elements are other possible strategies to change these energy gaps while increasing the photoluminescence quantum yield (PLQY) of the GQDs by suppressing the emitting traps.

In the top-down method, coal, which is considered the cheapest and easiest material to cleave compared to the other available materials, is exfoliated to form GQDs. For example, Ye et al. first sonicated coal in a mixture of concentrated sulfuric acid and nitrile acid for 2 h before heat-treating the mixture in an oil bath at 100–120 °C for 24 h to produce GQDs [48]. Yan et al. succeeded in controlling the band gap of the coal-derived GQDs using a surface functionalization technique. In the typical procedure, the coal-derived GQDs were mixed in a toluene solution with various organic compounds (e.g., o-phenylenediamine, 2,3-diaminonaphthalene, 1,8-diaminonaphthalene, 1,1′-bi(2-naphthylamine), p-anisidine, 4-(trifluoromethoxy)-aniline, or 4-(trichloromethoxy)-aniline) and then solvothermally treated at 180 °C for 12 h to systematically adjust the band gap of the GQDs [49]. Since there are many concerns about the use of strong concentrated acids, Shin et al. prepared GQDs from various natural carbon sources using an acid-free oxone oxidant-assisted solvothermal technique [50]. Another acid-free strategy based on the ultrasonic irradiation of a mixture of anthracite charcoal and N, N-dimethylformamide (DMF) was used by Zhang et al. to prepare GQDs [51]. Since most of the feedstocks used are non-renewable sources and sometimes special chemicals are required to obtain GQDs with tunable emissions, and since high temperatures and long reaction times are also required, the feasibility of the top-down method is significantly hindered, especially when addressing the current problem of global energy limitations.

In contrast, the bottom-up method uses polycyclic aromatic compounds or other molecules with an aromatic structure, such as fluorene [25,52,53,54,55]. Table 1 shows the characteristics of the top-down and bottom-up methods in the synthesis of GQDs. However, the toxicity of these aromatic precursors may have a negative impact on the environment and is therefore considered unsuitable for large-scale production. For this reason, many efforts have been made to utilize naturally available biomasses as the main starting materials for the synthesis of GQDs. Citric acid, as one of the most commonly used biomasses, can be pyrolyzed directly at 200 °C to obtain blue-emitting GQDs [56]. Nitrogen-containing molecules can be used, together with citric acid, to prepare readily nitrogen-doped GQDs (NGQDs). Wu et al. synthesized blue-emitting NGQDs with a PLQY of 36.5% from a mixture of citric acid and dicyandiamide using a hydrothermal technique at 180 °C for 3 h [57]. Recently, biomass waste has attracted much attention due to its low cost, renewable and environmentally friendly properties. Kumar et al. reported a one-step preparation of NGQDs from chitosan using a chemical vapor deposition (CVD) system at 250–300 °C [58]. To avoid the use of high temperatures, Chiang’s group used microplasma technologies to synthesize colloidal NGQDs with a PLQY of 30% from chitosan at ambient conditions [59]. Various strategies involving plasma flow, reaction time, and the type of acid used to dissolve chitosan were employed to control the functionalities and thus the energy gap of the resulting NGQDs [59,60]. Another promising biomass waste as a GQD precursor is lignin, which consists of phenyl skeletons and oxygenated branches [61]. Unlike simple structures, such as citric acid and glucose, both chitosan and lignin are biopolymers with complex structures, so the synthesis mechanism could involve a combination of top-down and bottom-up processes. The underlying mechanism is thought to consist of two main steps. These include the decomposition of long-chain structures into smaller units, the subsequent refusion into a nanograph domain, and the growth of GQDs [61,62]. Overall, the possibilities of the bottom-up method to utilize biomass derivatives as GQDs precursors have gained much interest nowadays, in order to realize a more sustainable, green, and eco-friendly approach to synthesize GQDs with unique properties and controlled structures to be usable for many applications.

### 2.2. Plasma Synthesis of GQDs

Plasma synthesis is considered one of the most popular gas-phase methods for the preparation of various GQDs, especially those with covalent bonding [70,71,72]. For example, QDs of germanium (Ge) and silicon (Si) have been synthesized using a conventional non-thermal plasma. In non-thermal plasma, factors, such as shape, surface area, quantum dot composition and size, can be controlled [73,74]. Plasma synthesis achieves doping, which is a major challenge for QDs [75,76,77]. GQDs synthesized by plasma usually take the form of powder, which can lead to surface modification. This can lead to excellent dispersion of QDs in water [78] or organic solvents [79] (i.e., colloidal quantum dots).

### 2.3. Method of Bioactivation

#### 2.3.1. Bioactive Carbon Sources

The development of bioactive materials for biomedical applications, such as inflammation therapy, is desirable if it is compatible with detectable properties and integrates efficient differentiation into biocompatible procedures. It has been possible to fabricate bioactive carbon dots (CD) with a size of about 4 nm, which have low toxicity, interesting safety responses, and unique photophysical properties. Bioactive CDs were prepared by a novel one-step hydrothermal method from aspirin and adenosine [80,81,82,83]. Multipurpose CDs are designed and fabricated using a bottom-up synthesis strategy to further manipulate chemical compounds and physical properties by introducing complex bioactive precursors, including nucleic acids, proteins, and small molecules. These bioactive CDs have different pharmacological activity from conventional citric acid-based CDs to expand their potential applications against pathogens and cancer [84,85].

#### 2.3.2. Biomass-Waste Derived GQD

**Figure 3:**
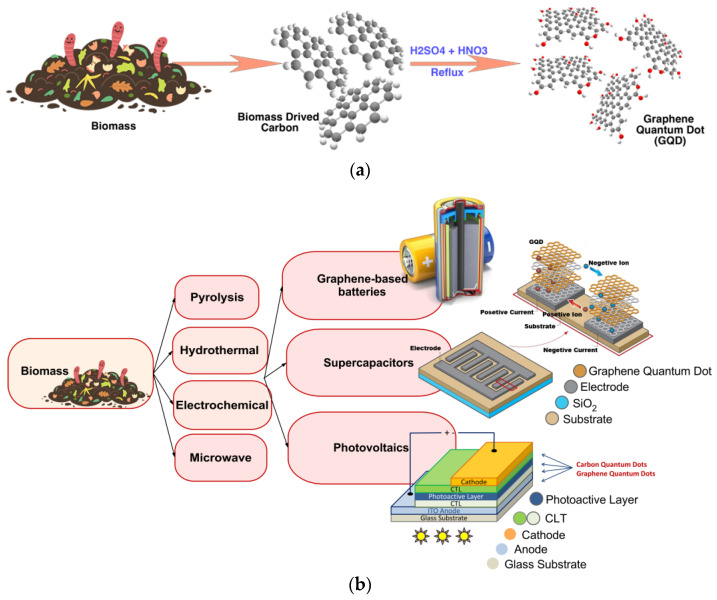
(**a**) The synthesis of GQD (C_57_H_26_O_11_) from different types of biomass-waste; (**b**) figure illustrates the various approaches used for converting biowaste into GQDs, along with how they can be used as energy sources.

Due to increasing customer demand, energy crisis and environmental degradation, scientists are looking for cost-effective, environmentally friendly, and easy ways to produce new advanced materials from renewable sources. Recently, graphene quantum dots (GQDs) have attracted much attention compared to other investigated materials, such as carbon-based nanomaterials, due to their attractive properties, such as low toxicity, long lifetime, high conductivity, good biocompatibility, and large surface area. Therefore, the properties of biomass waste-derived GQDs have been modified by adding surface inactivating agents and various functional groups through surface processing [31,86]. Kalita et al. investigated the modification of GQDs by amine functionalization to enhance the quantum efficiency of rice grain-derived GQDs. The results showed that the quantum efficiency was improved by 125% after amine functionalization, which was attributed to the superior electron donating ability of amine groups [31,86]. Table 2 shows the synthesis of GQD from different types of biomass waste. Figure 3a,b show GQDs obtained from biowaste and different approaches to convert biowaste into GQDs, with a description of their use as energy sources.

#### 2.3.3. Biologically Active Agents

Bioactive agents are factors that affect a living organ, cell, or tissue. They may be bioactive compounds, vitamins, drugs, phytochemicals, or enzymes. Bioactive agents used in biomedical devices and drugs can be contained in polymers [92]. The loading of bioactive agents in drug delivery systems is carried out by enzymatically reacting polymers and the cleavage of these agents by the target enzymes. The release of the therapeutic cargo occurs through the activation of the bioactive agents [93].

## 3. SERS GQD

**Figure 4:**
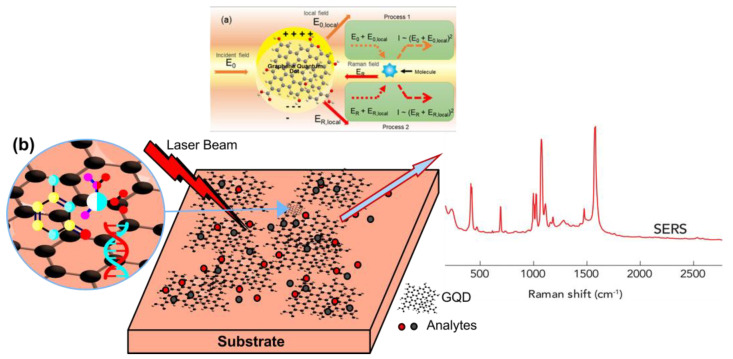
(**a**) Mechanism of the surface-enhanced Raman scattering (SERS) electromagnetic (EM) effect, electromagnetic SERS enhancement. Reprinted with permission from [114]. Copyright © 2020, American Chemical Society. (**b**) Schematic illustration of molecules on graphene and a substrate and Raman experiments. Reprinted with permission from [112], Copyright © 2010, American Chemical Society.

The frequency of the Raman peak of a phonon can be related to the chemical composition, the internal stress or surface state, and the shape and size of the GQD. Applications of Raman spectroscopy include the multiplexed detection of biomarkers from various compounds, controlling the synthesis of GQDs, studying vibrational properties associated with relaxation mechanisms, and analysing GQDs. For example, Raman spectra are associated with different concentrations of alloying constituents for alloyed GQDs, and this spectrum is also used to indicate the formation of quantum dots [94,95]. Therefore, the use of surface-enhanced Raman spectroscopy (SERS) is possible when the Raman intensity is undetectable and very low, such as the deposition of nanoparticles on a metal film, such as Ag, Al, or Au; these films have surface plasmon states with very limited electromagnetic fields that support emission and light absorption at the interface between air and metal. SERS also has applications from vibrational spectroscopy or photochemical studies [96] to the single emitter level [97,98], such as the detection of bacteria or impurities, and chemical analysis. Since different fabrication methods and many substrates have been proposed, it can be said that in the search for efficient and cheap metal substrates, SERS is a very active field. Examples are the gold nanoparticles recently obtained by the femtosecond exposure of a gold film [99], the strips of vertical or horizontal gold nanorods [100], the dual pyramidal gold nanoparticles [101], or the growth of silver nanoparticles on Si by the alternative method [100]. Hot spots occur when the key element is the presence of sharp points or dents on the surface of the metal substrate, where plasmonic states can be strongly confined. Hot spots are also crucial for future plasmonic applications in photochemistry or photovoltaics [96]. Some works have applied SERS to GQDs made of different materials: SnO_2_ [102], Si [103], CdS [104], PbS [105], or CdSe [106,107,108]. Thus, SERS is used as a promising technique for GQD detection; biomarker detection; in situ tracking of nanoparticle surface chemical properties, such as oxidation; and nanoparticle impact detection [26,103,105]. SERS in GQDs can be very useful because the material is not only environmentally friendly and compatible with the SERS mechanism but also has a large specific surface area, biocompatibility, and high chemical stability [109], which supports the improvement process of the mechanism for the improved detection of GQDs SERS. Although previous work SERS has reported GQDs in solid form, compared to GQDs in solution-based formats, it may require a complex process to use GQDs in solid form for other SERS applications. In addition, solution-based GQDs can be synthesized by a top-down approach, such as an electrochemical method that provides the ability to obtain SERS of GQDs in solution. This method uses the electric field as a mechanism to support the enhanced SERS detection of GQDs in the chemical mechanism (CM) to initiate the contact process of molecules and chemical reactions [110]. A schematic representation of the mechanism of SERS GQDs for analyte detection can be found in Figure 4. Graphene and other 2D materials have been developed for use as Raman enhancement substrates. This is due to their unique single sheet of carbon atoms in a 2D honeycomb crystal structure of electrons and phonons with one 2pz orbital of each sp2 hybridized carbon atom constituting a large, delocalized bond, forming an ideal flat surface and strong chemical interaction with many organic molecules [111]. As a SERS platform, graphene thus enables the independent investigation of the chemical enhancement mechanism (CM). Graphene may boost the Raman signals of molecules that have been adsorbed, and these substrates have shown to be promising for the detection of micro- and trace species. This impact was identified for the first time in 2010 by Ling et al. [112]. When graphene is treated with organic solvents, several “emerging bands” form on mechanically exfoliated graphene [113]. These “emerging bands” are scattered among the unidentified organic compounds included in the transparent tape used for graphene exfoliation. Graphene has a Raman amplification effect on trace residues. GQDs SERS is useful for both the fundamental investigations of SERS phenomena and several practical applications because of the graphene matrix’s significant benefits, such as homogeneity, repeatability, cleanliness, and low detection limit for aromatherapy dyes. Graphene makes SERS applications of Raman-enhanced substrates more quantitatively controlled.

## 4. Detection Mechanisms of SERS

**Figure 5:**
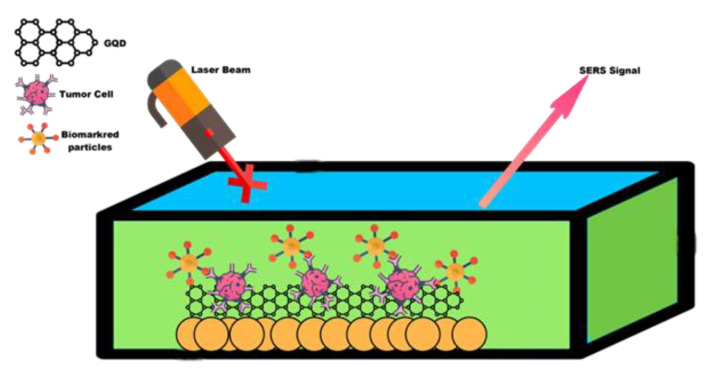
In vivo imaging of SERS-enhanced GQDs for detection of tumours, inflammation and living tissues.

Since the discovery of SERS, several mechanisms have been proposed, but only two are widely accepted today: the electromagnetic theory (EM) and the chemical amplification theory (CE). The EM theory is more dominant because it can amplify the Raman signal up to ten thousand times. While CE amplifies the Raman signal up to 100 times. In the EM model, the laser interacts with the metal surface. As a result of this interaction, dissolved surface plasmons are stimulated, which amplify the field near the surface. In the CE theory, the electron states of the adsorbent change due to chemical adsorption. In the SERS phenomenon, both factors occur simultaneously, which is why the Raman signal can be amplified to such an extent. In general, surface-enhanced Raman spectroscopy is very similar to Raman resonance spectroscopy. The difference being that the resonances present are not exclusively of the intramolecular type. Surface-enhanced Raman spectroscopy or SERS is also a method of Raman spectroscopy that has very high sensitivity in deciphering materials [115,116,117]. This method has numerous applications in medical science. Since it does not damage natural tissues, it does not require sample preparation and is very rapid. Therefore, this method is used to detect proteins in body fluids. It is also used for diagnosis and treatment of tumours and cancer, the treatment of neurological diseases, the detection of COVID-19, and the detection of coronavirus RNA. This technology for detecting urea and label-free plasma in human serum can be useful in cancer screening. SERS can be used for drugs, forensics, the detection of drugs and explosives, the study of redox processes at the single molecule level, the quantitative analysis of small molecules in human biological fluids, the quantitative detection of biomolecular interactions and more. In a tumour testing, the tumour is grown in vitro. In reality, the test is performed on living tissue (these tests are called in vivo tests). SERS can be used to detect low molecular weight biomolecules (Figure 5) [118,119,120,121]. Moreover, the mechanism of SERS detection in the efficiency of GQDs due to surface factorization and heteroatomic doping has been discussed in few studies. Therefore, in the near future, in-depth studies on these topics, which open a new window to developing highly effective improved GQDs SERS, will help scientists and researchers to understand the inflammatory diagnosis of GQDs. Finally, low-cost industrial production is urgently needed to develop and expand these approaches in the near future. To diagnose the inflammation and interior of GQDs and control the macroscopic properties of GQDs, one must be able to produce GQDs by controlling their size as much as possible [122].

## 5. Inflammatory Biomarkers

The term biomarker was first used by the National Institutes of Health in the United States in 1980 [123]. Biomarkers are a new method in medicine in which these markers measure specific indicators and examine routine biological processes, pathological processes, or pharmacological responses through therapeutic or health mediators. Specific RNA/DNA gene sequences, antibody determinations, and organic metabolite measurements are also identified [124,125,126]. For example, blood pressure is a biomarker for stroke risk and glucose levels are a biomarker for patients with diabetes; cholesterol levels are also used to determine cardiovascular disease risk [127]. For the nervous system, muscle, blood, nerve, cerebrospinal fluid, skin, and urine have been used to extract information from the brain in healthy and unhealthy states. These tools and technologies directly measure biological factors (such as blood or CSF) or they work together with brain imaging to measure changes in the composition, function, and structure of the nervous system. Biomarkers are classified according to the sequence of events from “exposure” to “disease”. Biomarker “exposure” is used to predict hazards and to establish a link between external exposures and internal dosimetry. Disease biomarkers are used for the screening, identification, and monitoring of disease progression [128,129,130]. In addition, these biomarkers can be used to refine drugs to improve therapeutic outcome and health [127]. A good biomarker must be more than 80% specific and have an equally high sensitivity (above 80). The role of biomarkers is not only identification, but they also have the potential to be predictive or play a role in the development of new therapies [131]. Plasma biomarkers are divided into pro-inflammatory biomarkers that include the following subgroups [132]:

### 5.1. C-Reactive Protein and Cytokines

**Figure 6:**
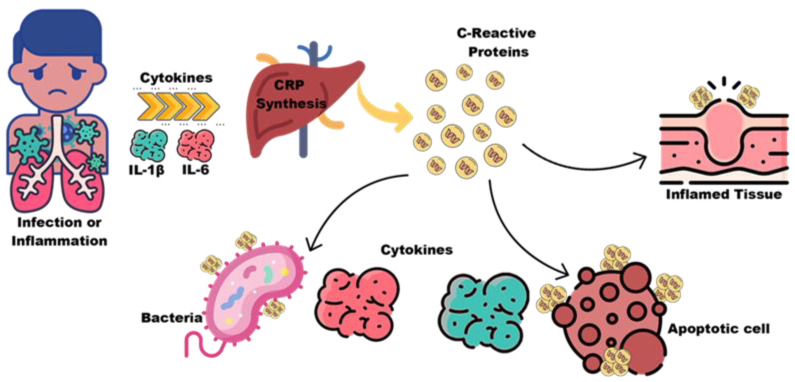
Schematic of C-reactive protein and cytokines the functional pathways of CRP.

C-reactive protein (CPR) is a protein involved in host safety and is mainly released by adipose tissue and liver in response to inflammatory stress. On the other hand, its reaction with the crystallisable receptor fragment leads to the production of pre-inflammatory cytokines. According to studies, CRP levels increase in patients with migraines and in women who suffer migraines with aura [132,133,134]. Cytokines are small proteins that are released by the stimulating neuropeptides involved in migraines. Therefore, their serum levels increase during migraine attacks, for example [132]: Tumour necrosis factor alpha (TNF-α), as a proinflammatory cytokine, plays a key role in the regulation of immune cells; it is also involved in clot formation, cell proliferation, apoptosis, lipid metabolism, and increases in plasma after migraine attacks. Transforming growth factor beta 1 (TGF-β1), a proinflammatory cytokine, also has several functions. This type of cytokine not only plays an important role in immune system function and blood vessel formation, but also causes motility, apoptosis, cell growth control, and differentiation [132,134,135]. TGF-β1 levels are increased in migraine patients compared to controls [133], but there is no difference between levels in aura and without aura. Fatigue and lack of energy during migraines are due to increased TGF-β1 levels [136]. Figure 6 shows a schematic of C-reactive proteins and cytokines, the functional pathways of CRP. As a result of cytokines, such as IL6 and IL1*, hepatic CRP expression increases significantly. CRP circulates, opsonizing bacteria and apoptotic cells so they can be cleared through the complement system. Immunomodulatory cytokines, such as IL10, may be released by phagocytic cells in response to CRP ligation. Studies have found that plasma CRP deposited onto inflamed tissue breaks down into biologically active monomeric subunits, which can be credited with proinflammatory effects.

### 5.2. Adiponectin and Lipids

**Figure 7:**
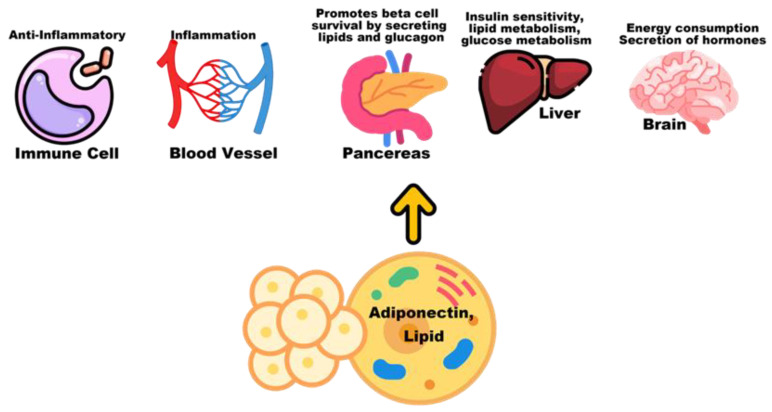
The major processes through which adiponectin maintains metabolic homeostasis.

Adiponectin is released from adipose tissue and is an anti-inflammatory cytokine, like IL-10, that inhibits the expression of pre-inflammatory cytokines. It plays an important role in regulating glucose homeostasis and other metabolic processes and is associated with obesity and BMI [132,133]. Research shows that lipids are associated with high cholesterol and migraine. In addition to total cholesterol, there is evidence that people with migraine have further increases in lipid subtypes, such as low-density lipoprotein cholesterol, oxidised LDL-c, triglycerides, and also a decrease in the anti-inflammatory high-density lipoprotein cholesterol [132,137]. Figure 7 shows the main processes by which adiponectin maintains metabolic homeostasis.

### 5.3. Raman Spectrum of the Inflammatory Biomarkers

Recently, biomarker detection based on Raman spectrum technology has been widely and comprehensively developed. Raman spectrum technology has attracted more attention with the rapid development of nanotechnology. Raman spectrum technology plays an important diagnostic role from organelle functionality, inflammation detection, and virus detection to cell activity detection. However, there are still many challenges in the development of Raman spectrum technology [138,139]. Monitoring different types of inflammation in the early stages by in vitro diagnosis is vital. For early detection and prognosis of inflammation, biomarkers, such as proteins, miRNAs, DNAs, and other biomolecules, must be evaluated [140,141]. The specificity and sensitivity of the Raman spectrum make it possible to accurately detect related physiological analytes in complex biological fluids. To detect inflammatory biomarkers, the Raman spectrum is linked to relevant detection molecules (such as antibodies and aptamers) to allow specific targets to be measured with Raman signals [142,143].

## 6. Detection of Inflammatory

**Figure 8:**
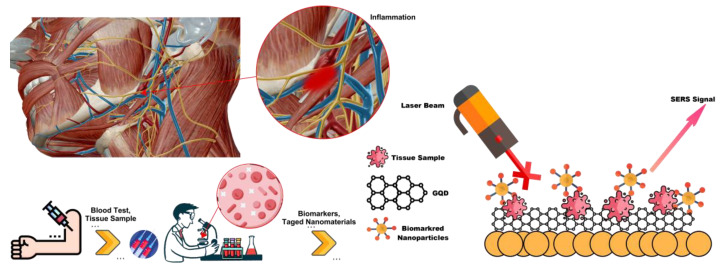
Detection of inflammatory by SERS-enhanced GQDs.

The immune system is involved in the development of a variety of diseases [144,145,146]. The regulation of immune responses by direct tissue imaging in diseases, such as atherosclerosis [147], rheumatoid arthritis [148], malaria [149], and stroke [150], is due to the increasing interest in understanding the molecular and cellular interactions of the pathway. A number of intravital microscopy techniques have been used to study these interactions. For example, the two-photon fluorescence microscope (TPM) has become the method of choice for stimulating fluorophores deep within tissues due to its unique ability to produce light in the near-infrared range. However, successful imaging is limited to depths of a few hundred micrometres [151]. In addition, fluorescence imaging produces a broad emission spectrum [152] that often leads to photobleaching [153], as this imaging generally suffers from poor discrimination between specific fluorophores and background autofluorescence beyond a small optical window [154]. One way to enhance the Raman signal is to use the SERS method. This method involves vibrational spectroscopy in which molecules are adsorbed onto a metal surface with a nano-sized surface area. SERS-activated GQDs were encoded with a unique Raman signal that was monitored under a wide range of excitations and conditions. GQDs containing active Raman molecules were conjugated with specific monoclonal antibodies against intercellular adhesion molecule 1 (ICAM-1) to detect early-stage inflammation. The non-invasive measurement of ICAM-1 expression by SERS is possible in vivo with double the sensitivity of double photon fluorescence [155]. Therefore, a new approach for the diagnosis of inflammation in vivo using GQDs SERS was considered. Using a metal surface to enhance Raman scattering from molecules located near or attached to the surface results in vibrational spectroscopy called SERS. A wide range of different surfaces and metals can be used to achieve this goal. GQDs, however, offer a great format. By binding molecules with a unique and strong Raman spectrum, called Raman reporters, to GQDs and encapsulating them in a silicon-containing shell, GQDs with improved SERS are produced. The Raman reporter molecules are protected by a silica shell that acts as a coating and gives the GQD a unique and strong SERS signal [156,157]. In vivo imaging of SERS-enriched GQDs has also been used to monitor inflammation and reuse. Although this is not a disease process, it is still important as any changes may indicate infection and non-hidden disease states Figure 8 [155].

## 7. Perspectives

The multiple applications of Raman and the significant improvement of GQDs SERS for the diagnosis of inflammatory diseases are direct consequences of the numerous advantages they offer. Unlike their fluorescent counterparts, they produce specific, sharp molecular spectra that give these techniques immediate and easy access to multiple ways of diagnosing disease. In addition, there is the possibility of combined Raman and SERS imaging of tissues and cells, so that biochemical information and features of the inflammatory process can be obtained simultaneously as active SERS nanotags are formed. To improve GQDs SERS and remain at the forefront of inflammatory disease diagnosis, further studies in physiological representative media, clinical samples, and in vivo are required. Once these programmes have fully demonstrated their reproducibility, sensitivity, robustness, repeatability and selectivity in vivo, the laboratory will be transferred to a clinical setting. Once these applications have fully demonstrated their techniques, sensitivity, and reliability they will be strong contenders that will revolutionise our ability to diagnose inflammatory diseases. Ultimately, the hope is that the use of GQDs, which SERS enhance for the in vivo diagnosis of inflammation, will be part of a growing toolkit for next-generation non-invasive imaging and in vivo diagnosis.

## 8. Conclusions

This review summarises recent advances in the diagnosis of inflammation using graphene quantum dot SERS. Three subtopics are described, including the method of GQD synthesis, the method of bioactivation, inflammatory biomarkers, the plasma synthesis of GQDs and SERS GQD, and the detection mechanisms of SERS and the detection of inflammation. There are key points in the development of SERS GQD and its biomedical applications, as the rapid evolution of SERS GQD from biological to biomedical applications has been remarkable over the past decades. Significant progress has been made in improving diagnostic sensitivity and multiplexity. Recent advances have led to SERS GQD being used to diagnose inflammation in necrotic tissue and damaged cells, which will be of great importance for use in medical facilities. In short, SERS GQD has the advantageous properties of unprecedented multiplexing capability, perfect signal specificity and high sensitivity. Therefore, there are driving forces to exploit these properties for important applications. However, there are still some steps to be taken when using SERS GQD for clinical applications.

## Figures and Tables

**Table 1:** Characteristics of top-down and bottom-up methods in the synthesis of GQDs.

	Subgroup	Initial Material	Size (nm)	Quantum Efficiency	Ref.
Top-down	Acid oxidation	Carbon black	15	44.5	[63]
	Hydrothermal	Graphene oxide	5–13	5	[64]
	Solvothermal	Graphene oxide	3–5	1.6	[65]
	Microwave	Graphene oxide	2–7	8	[66]
	Ultrasound waves	Graphene	3–5	-	[54]
	Electrochemical	Graphite	5–10	-	[67]
Bottom-up	Pyrolysis of the precursor	Glucose	1.65–21	-	[68]
	Catalytic opening of the cage	Fullerene 60	2.7–10	15–30	[46]
	Pyrolysis	Hexa benzo chromen	~60	-	[69]

**Table 2:** Synthesis of GQDs from different types of biomass-waste.

Precursor	Product	Preparation Approach	Size (nm)	Ref.
Rice grains	GQDs	Pyrolysis	2–6.5	[87]
Fenugreek leaf extract	GQDs	Pyrolysis and hydrothermal treatment	3–10	[88]
Wood charcoal	GQDs	Electrochemical oxidation	3–6	[89]
Neem leaves	GQDs, Am-GQDs	Pyrolysis and hydrothermal treatments	5–6	[90]
Coffee grounds	GQDs, PEIGQDs	Hydrothermal treatment	1.88 (GQDs)2.67 (PEIGQDs)	[91]

## Data Availability

All data generated or analysed during this study are included in thepublished article.
